# Data from a proteomic analysis of colonic fibroblasts secretomes

**DOI:** 10.1016/j.dib.2014.08.003

**Published:** 2014-08-21

**Authors:** Sun-Xia Chen, Xiao-En Xu, Xiao-Qing Wang, Shu-Jian Cui, Lei-Lei Xu, Ying-Hua Jiang, Yang Zhang, Hai-Bo Yan, Qian Zhang, Jie Qiao, Peng-Yuan Yang, Feng Liu

**Affiliations:** Department of Medical Systems Biology of School of Basic Medical Sciences and Institutes of Biomedical Sciences, Fudan University, 131 Dongan Road, Shanghai 200032, China

**Keywords:** Tumor microenvironment, Colorectal cancer, Cancer-associated fibroblast, Proteomics, Secretome, Follistatin-related protein 1

## Abstract

The tumor cell proliferation, migration and invasion were influenced by the interaction between the cancer cells and their microenvironment. In current study, we established two pairs of the primary fibroblast cultures from colorectal adenocarcinoma tissues and the normal counterparts and identified 227 proteins in the colonic fibroblast secretomes; half of these proteins were novel. The mass spectrometry data and analyzed results presented here provide novel insights into the molecular characteristics and modulatory role of colon cancer associated fibroblasts. The data is related to “Identification of colonic fibroblast secretomes reveals secretory factors regulating colon cancer cell proliferation” by Chen et al. [1].

**Specifications table**

**Table:** 

Subject area	*Biology*
More specific subject area	*Cancer microenvironment*
Type of data	*Excel tables*
How data was acquired	*Mass spectrometry*, *data acquired using Synapt G1 mass spectrometer*
Data format	*Analyzed*
Experimental factors	*Conditioned media were collected*, *proteins were concentrated using filters*
Experimental features	*The proteins were separated using SDS-PAGE*, *in-gel tryptic digested and analyzed using LC-MS*
Data source location	*Shanghai*, *China*
Data accessibility	*The data is available with this article and is related to*[1]

**Value of the data**

• 227 colonic fibroblast secretome proteins are identified at a false discovery rate of 1.3%.
• 125 proteins (55.1%) are novel identified secretome proteins of colonic fibroblasts.
• These proteins are enriched for functional categories of extracellular matrix, adhesion, cell motion, inflammatory response, redox homeostasis and peptidase inhibitor.
• The data are valuable for understanding of the molecular feature of colonic fibroblasts and are ripe for further exploration and data mining.
• The data are useful for comparing purpose when addressing the heterogeneities of the fibroblast expressions from different isolation.

## 1 Data, experimental design, materials and methods

Secreted proteins were extracted from the conditioned medium of one pair of the fibroblast cultures. We performed a proteomic analysis of the proteins using a Synapt G1 mass spectrometer. The mass data interpretation was performed using ProteinLynx Global Server, X! Tandem and Scaffold softwares. The proteomic data presented here include the protein and spectrum identification results, as well as the cellular component annotation and functional analyzing results. 227 proteins were identified with a false discovery rate of 1.3% based on 15,000 assigned spectra. Half of the proteins were novel identifications in the secretome of colonic fibroblasts comparing with the known report. The subcellular localization of the identified proteins were annotated using UniProt database, DAVID, SignalP, SecretomeP, Phobius, WoLF PSORT and exosome databases. The occurrence of the fibroblast secretome proteins in the colon cancer cell proteomes was also analyzed. The functional enrichment of the identified proteins was performed using DAVID.

## 2 Establishment of fibroblast cultures from fresh surgical specimen

We established two pairs of colon cancer-associated fibroblast (CAF) and normal fibroblast (NF) cultures. The fresh colorectal cancer tissues and adjacent normal colonic tissues (at least 5 cm away from the loci of cancerous tissue) from two patients with colon ulcerated adenocarcinoma were collected during surgery at the Zhongshan Hospital of Fudan University according to the procedure described in the Journal of Proteomics paper [1]. The clinic and pathological data of the two patients are available in Supplementary Table 1.

## 3 Proteomic analysis of the colonic CAF and NF secreted proteins (SPs)

Fifty micro gram of the SP extracted from the 1031_NF and 1031_CAF conditioned medium (CM) were separated using 10% SDS-PAGE. Equal amount of sample was loaded in triplicate. Two gel lanes of each sample were cut into 10 slices separately and subjected to in-gel tryptic digestion according to an optimized procedure [2]. The digested peptides were analyzed using Synapt G1 mass spectrometer as described previously [3]. Briefly, the peptide mixture from each gel slice was first loaded onto a reverse phase (RP) trap column (C18, 5 μm, 180 μm×20-mm Symmetry C18 nanoAcquity column, Waters) for on-line desalting at a flow rate of 10 μL/min. The peptides were then eluted from the trap column into an analytical fused silica nanoAcquity UPLC column of 75 μm×200-mm inner diameter packed with C18 of 1.7-μm pore diameter stationary phase (Waters). The column oven temperature was maintained at 40 °C. Mobile phase A contains 0.1% formic acid (FA) in water, while mobile phase B was 100% acetonitrile (ACN)/0.1% FA. The mobile phases were delivered using a nanoAcquity UPLC system (Waters) at a flow rate of 250 nL/min. The peptides were separated with a linear gradient of 3–15% mobile phase B (5 min), 15–35% B (65 min), 35–98% B (5 min), 98% B (5 min), and 98–3% B (10 min). The peptides were ionized at the end of a 10-μm-inner diameter PicoTip nanospray emitter (New Objective) which was connected to the end of the analytical column. A voltage of 3200 V was applied to the emitter for a steady spray. The ions got into a Synapt G1 mass spectrometer (Waters) via a Nano electrospray ionization (ESI) source. The source-inside temperature was maintained at 100 °C. During the data acquisition, a reference sprayer using 320 fmol/μL lock mass compound [Glu1]-fibrinopeptide B (GFB) (Sigma) was sampled with a frequency of 30 s at a flow rate of 200 nL/min. The precision of the mass data was calibrated using the doubly charged monoisotopic ion of GFB. The MS^E^ data was acquired using alternating low-energy collision at 6 eV for survey scan and high-energy collision ramped from 15 to 55 eV for tandem MS. The spectral acquisition time in each mode of 1.2 s and a full cycle was 2.44 s. The mass range of the survey scan was set at *m*/*z* 100 to 1800.

## 4 MS data interpretation by database (DB) searching

ProteinLynx Global Server (PLGS) v2.5 (Waters) was used for peak picking and DB searching as described previously [3]. The searching DB contains 20,273 human protein entries extracted from UniProtKB/Swiss-Prot Release 2014_01. The DB searching result files in zip format were exported from PLGS using a Scaffold plugin and were parsed into Scaffold v4.2.1 (Proteome Software, Inc.). For data integration and validation by Scaffold, the standard legacy PeptideProphet scoring system [4] and the standard experiment wide protein grouping method were adopted. An integrated version of X! Tandem (Version: CYCLONE (2010.12.01.1)) in Scaffold was used for an additional DB searching using the same parameters as PLGS. The searching results from both PLGS and X! Tandem were combined automatically by Scaffold. Protein identifications were accepted with a protein probability >99.0% assigned by ProteinProphet [5] and ≥3 unique peptides of <0.1% false positive rate (FDR) assigned by PeptideProphet algorithm [4]. The protein abundance was represented by the exclusive spectrum count which was normalized across different samples. Supplementary Table 2 contains the proteomic analysis and the bioinformatics analysis results of the identified proteins. The table lists the protein name, gene name, gene symbol and molecular weight. The proteomic data include the exclusive spectrum count, exclusive unique peptide count, percent coverage, exclusive unique spectrum count and the total spectrum count. These results were output from Scaffold. The subcellular localization analysis results include the information or prediction from UniProt, SignalP, Phobius, SecretomeP, WoLF PSORT, DAVID, ExoCarts and Vesiclepedia. Based on these analyses, a final annotation was made manually. Tissue specificity gene expression enrichment was analyzed using the Gene Enrichment Profiler and a smooth muscle-enriched expression might indicate the characteristics of myofibroblast. A comparison to the identification results of De Boeck et al.׳s was made [6]. We also compared the identified fibroblast protein with known colon cancer proteins reported by 10 articles. The first paper by Meike et al. analyzed the expressions of colon cancer cell lines Caco-2, HT-29, HCT-116, SW480, SW1398 and identified 2361 proteins [7]. The other papers containing colon cancer cell identifications are listed in Table 1. The GO analysis results, including the biological process, cellular component and molecular function, were generated using Scaffold. We downloaded the GO annotation file for human gene (ftp://ftp.ebi.ac.uk/pub/databases/GO/goa/HUMAN/gene_association.goa_human.gz) on February 6, 2014 and used this GO file for annotation using Scaffold.

The spectrum identification results are deposited in Supplementary Table 3, including the ion *m*/*z*, peptide sequence, PLGS score, X! Tandem score, charge, intensity, etc. The Supplementary Table 3 is an output from Scaffold using “export” function for spectrum report with minor editing. Statistics on the spectrum and protein identification reported by Scaffold is summarized in Supplementary Table 4. It should be noted that the “Show lower scoring matches” of Scaffold was disabled when reporting and exporting the identification results.

The spectra of eight representative proteins identified in the fibroblast secretomes are shown in Supplementary Fig. 1, together with an illustration of the sequence coverage of each protein across different proteomic analyses. The spectra and the sequence coverage illustrations were extracted from the Scaffold results.

## 5 Subcellular localization analysis of the identified proteins

To get a precise prediction, the subcellular localization analysis was performed using multiple methods. The cellular component annotations by Gene Ontology (GO) of the identified proteins were extracted from the flat text file retrieved from UniProt DB (hwww.uniprot.org). Similarly, the GO annotation of the cellular component was analyzed using the DAVID Bioinformatics Resources (http://david.abcc.ncifcrf.gov/). SignalP v4.1 (http://www.cbs.dtu.dk/services/SignalP/) and Phobius (http://phobius.sbc.su.se/) were chosen to analyze the classical SP. Proteins longer than 6000 amino acids were excluded from analysis by SignalP. Non-classical SPs were predicted using SecretomeP v2.0 (http://www.cbs.dtu.dk/services/SecretomeP/), with a length restriction of less than 4000 amino acids. Additionally, the subcellular localization was predicted using WoLF PSORT (http://wolfpsort.seq.cbrc.jp/). Potential exosome protein were revealed by comparing the protein names and accessions with those registered in ExoCarta DB version 4.1 (http://www.exocarta.org) [17] and Vesiclipedia DB version 2.1 (http://www.microvesicles.org/) [18]. All of these information were integrated in Supplementary Table 2 and manually checked and a final annotation was made for each protein.

## 6 RNA interference (RNAi) and ectopic expression

To address the functionalities of the identified proteins, we silenced the coding genes of these proteins in a normal colonic fibroblast cell line, CCD-18Co, and then performed coculture assays with colon cancer cells. Follistatin-related protein 1 (FSTL1) was chosen for ectopic expression analysis in CCD-19Co, which was used for coculture assays. The small interference RNAs (siRNAs) and cloning primers are listed in Supplementary Table 5.

## 7 Functional enrichment analysis of the identified proteins

GO enrichment analysis was performed using DAVID and significant enrichment categories were accepted with *p* value and Benjamini value less than 0.05. The enrichment analysis results are shown in Supplementary Table 6. Tissue specificity gene expression enrichment was analyzed using the Gene Enrichment Profiler (http://xavierlab2.mgh.harvard.edu/EnrichmentProfiler/). The results for tissue expression enrichment are embedded in Supplementary Table 2.

## 8 Comparison with the known identifications of colon cancer cells

The identified fibroblast proteins were compared with a batch of known proteomic analyses of colon cancer cells to evaluate their specific expression properties [7–16]. The identified proteins of these reports varied from ~40 to 2300. The comparison was mainly based on official gene symbols. In cases when needed, the International Protein Index (IPI) accessions were mapped to gene symbols and UniProt accessions using the Protein Identifier Cross-Reference (PICR) (http://www.ebi.ac.uk/Tools/picr/). The analyzing results were integrated in Supplementary Table 2. Area-proportional Venn diagram was generated with BioVenn (http://www.cmbi.ru.nl/cdd/biovenn/index.php).

## Figures and Tables

**Table 1:** Colon cancer proteomic identifications used for comparison.

No.	Colon cell lines analyzed	Protein identified	Reference
1	Caco-2/HT-29/HCT-116/SW480/SW1398	2361	[7]
2	CaCo-2/HCT-GEO	176	[8]
3	SW480/SW620	1929	[9]
4	LIM1215	56	[10]
5	SW480/SW620	910	[11]
6	LIM1215	136	[12]
7	SW620	234	[13]
8	KM12C/KM12SM	2088	[14]
9	Colo205/SW480/SW620	2042	[15]
10	SW480/SW620	1675	[16]

## Footnotes

Appendix A Supplementary data associated with this article can be found in the online version at doi:10.1016/j.dib.2014.08.003.
