# Supplementary material for: Data from a proteomic analysis of colonic fibroblasts secretomes
Source: Data Brief. 2014 Aug 21;1:19–24. doi: 10.1016/j.dib.2014.08.003 (PMC4459868; doi:10.1016/j.dib.2014.08.003)
Supplement: Supplementary file 1 — Supplementary Data [file mmc1.pdf]

Supplementary Figure 1

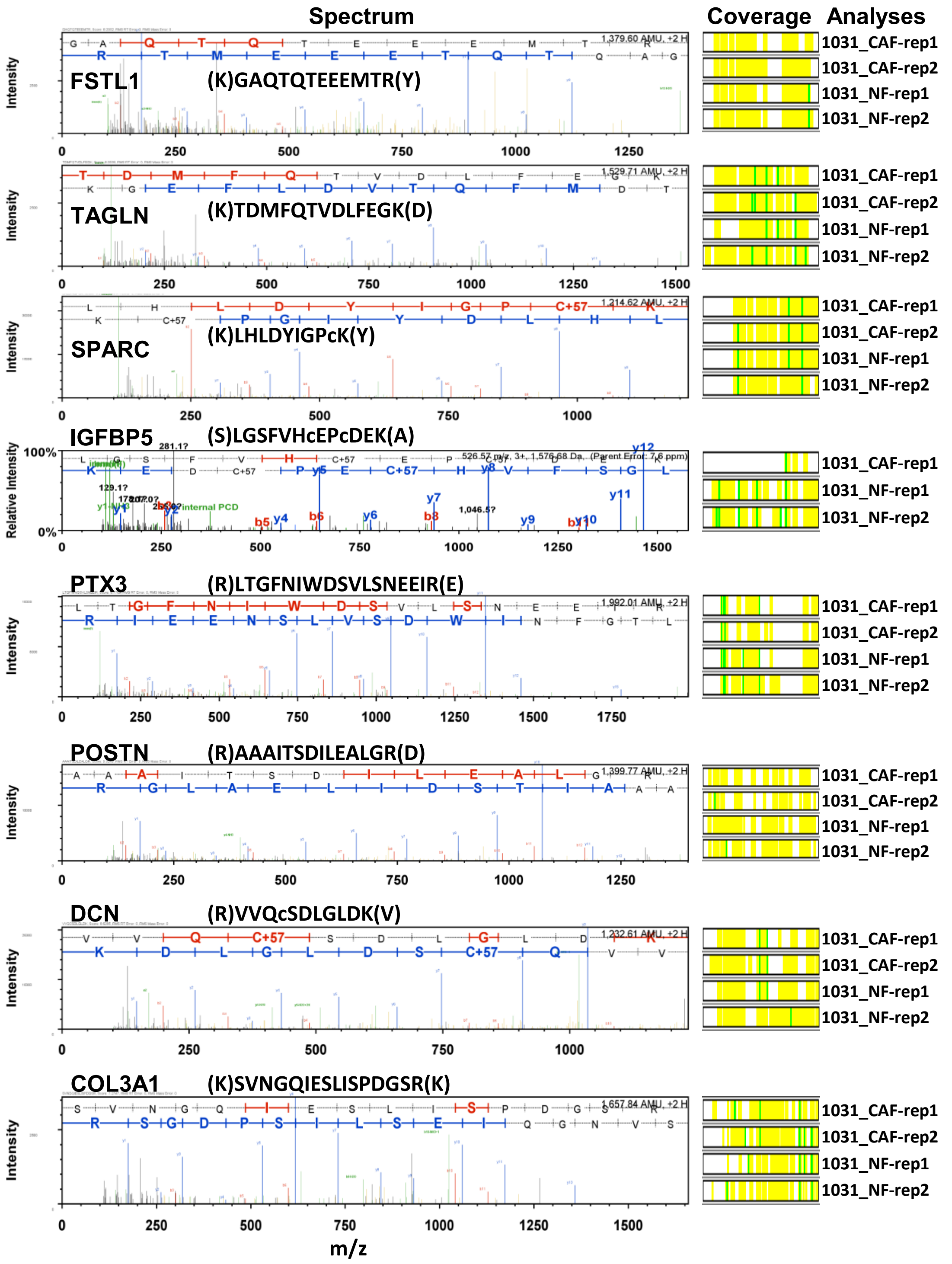

Supplementary Figure 1. **Representative tandem mass spectra of the secreted proteins in the fibroblasts.** Sequential *b* and *y* ions are highlighted in blue and red, respectively. Colored boxes visually depict the fraction of the amino acids identified in each protein (Coverage) across different replicated MS (Analyses). Identified amino acid and post-translational modification (PTM) are colored yellow and green, respectively.
